# Mediastinal teratoma presenting with hemoptysis and pleuritis misdiagnosed as tuberculosis (empyema)

**DOI:** 10.1186/s12887-018-1357-7

**Published:** 2018-12-04

**Authors:** Jinrong Liu, Baolin Tian, Qi Zeng, Chenghao Chen, Chunju Zhou, Huimin Li, Yuelin Shen, Shunying Zhao

**Affiliations:** 1 Department of Respiratory Medicine, Beijing Children’s Hospital, National Center for Children’s Health, Capital Medical University, Nanlishi Road 56, Xicheng District, Beijing, People’s Republic of China; 2 Department of thoracic surgery, Beijing Children’s Hospital, National Center for Children’s Health, Capital Medical University, Nanlishi Road 56, Xicheng District, Beijing, People’s Republic of China; 3 Department of Pathology, Beijing Children’s Hospital, National Center for Children’s Health, Capital Medical University, Nanlishi Road 56, Xicheng District, Beijing, People’s Republic of China

**Keywords:** Teratoma, Pancreatic tissue, Hemoptysis, Pleuritis, Children

## Abstract

**Background:**

Mediastinal teratoma is uncommon in children. It can be very difficult to diagnose especially in early stage. Rarely, teratoma may rupture into adjacent structures and lead to lung lesions or pleuritis. The main rarity of our reported cases was the dynamic imaging findings very similar to the developmental process of tuberculosis in patients 1 and 2, the pachypleuritis in patients 2 and 3, the extremely elevated inflammatory markers very similar to empyema in patient 3, and the extremely atypical tumor shape in all patients.

**Case presentation:**

We present three pediatric patients presenting predominantly with recurrent hemoptysis and/or chest pain who were ultimately diagnosed with mediastinal teratoma containing pancreatic tissue. All three patients were initially suspected to have tuberculosis or empyema, and underwent relevant treatment, but without improvement. Patient 1 had left hilar enlargement, and subsequently an enlarging calcified cavity within high-density consolidation was identified. Patient 2 initially presented with right-sided pulmonary consolidation and pleuritis, and subsequently developed right lower lobe calcification, pleural thickening, and irregular soft tissue in the right inferior mediastinum. Patient 3 was initially found to have right lobe consolidation accompanied by a massive right-sided pleural effusion with extremely elevated inflammatory markers in serum and pleural effusion. The effusion later acquired heterogeneous density and appeared to become encapsulated. In patients 2 and 3, pleural biopsy identified fibrous tissue (with and without granuloma). Thoracotomy/thoracoscopy revealed mediastinal teratoma in each case, all of which were completely excised and the patients made uneventful recoveries. Histopathologic analysis revealed mature cystic-solid teratoma containing pancreatic tissue in all patients, and calcification in patients 1 and 2.

**Conclusions:**

Clinicians should be mindful that mediastinal teratoma is a potential cause of hemoptysis, lung lesions and pleuritis. Calcification and pachypleuritis on chest imaging especially in patients without fever should be highly suspected of mediastinal teratoma. Pleural biopsy sometimes fails to assist in making a definitive diagnosis.

## Background

Mediastinal teratoma is unusual in children. It is often difficult to diagnose because of its few early symptoms. Rarely, a cystic teratoma may rupture into adjacent structures, such as the pleural space, pericardium, lung parenchyma or tracheobronchial tree [1–6], however tumor shape of the previous reported cases was more relatively typical than our present cases.

Here, we report a series of three children with recurrent hemoptysis and pleuritis attributed to mediastinal teratoma containing pancreatic tissue (all three patients) and calcification (patients 1 and 2). All these patients were initially suspected to have pneumonia, tuberculosis or empyema, and underwent relevant treatment, but without improvement. The main rarity of these cases was the dynamic imaging findings similar to the developmental process of tuberculosis in patients 1 and 2, the pachypleuritis in the patients 2 and 3, the extremely elevated inflammatory markers in serum and pleural effusion similar to empyema in patient 3, and the extremely atypical tumor shape on chest imaging in all patients especially in patient 2.

### Case presentation

#### Case 1

**Fig. 1:**
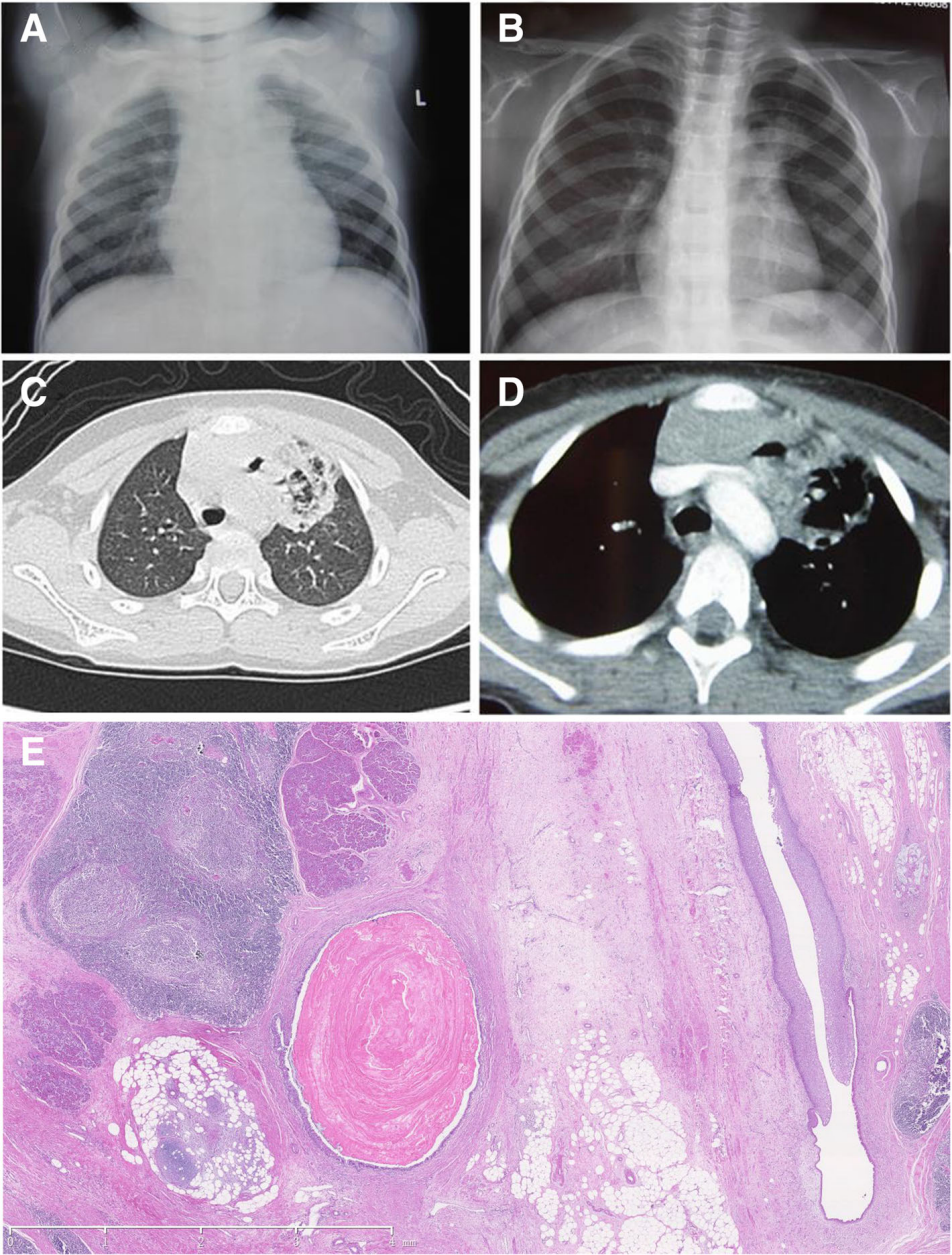
Chest X-ray/lung CT showing the presence of 1**a** left hilar enlargement, 1**b** an emerging cavity with high-density consolidation, 1**c-d** high-density opacities occupying the left upper lobe, and consolidation with cavitation and calcification adjacent to the mediastinum. Histopathologic analysis (× 200) revealed mature pancreatic tissue, gastrointestinal epithelium, cartilage tissue, and sebaceous material within the mass (Fig. 1**e**)

A 5-year-old boy presented with a 9-month history of recurrent hemoptysis and mild wet cough. Chest X-ray revealed left hilar enlargement (Fig. 1a), and subsequently an emerging cavity within high-density consolidation (Fig. 1b). He was treated for tuberculosis for 5 months, but his hemoptysis (2–10 ml of blood each time) became worse. On admission to our hospital, contrast-enhanced computed tomography (CT) revealed high-density opacities occupying the left upper lobe, and consolidation with cavitation and calcification adjacent to the mediastinum (Fig. 1c-d). We considered an atypical intrapulmonary tumor or malformation, and performed an open thoracic exploration for a definitive diagnosis. Exploration revealed a thymic mass tightly adherent to the left lung, which was partially eroded. The left upper lobe and tumor were excised completely.

#### Case 2

**Fig. 2:**
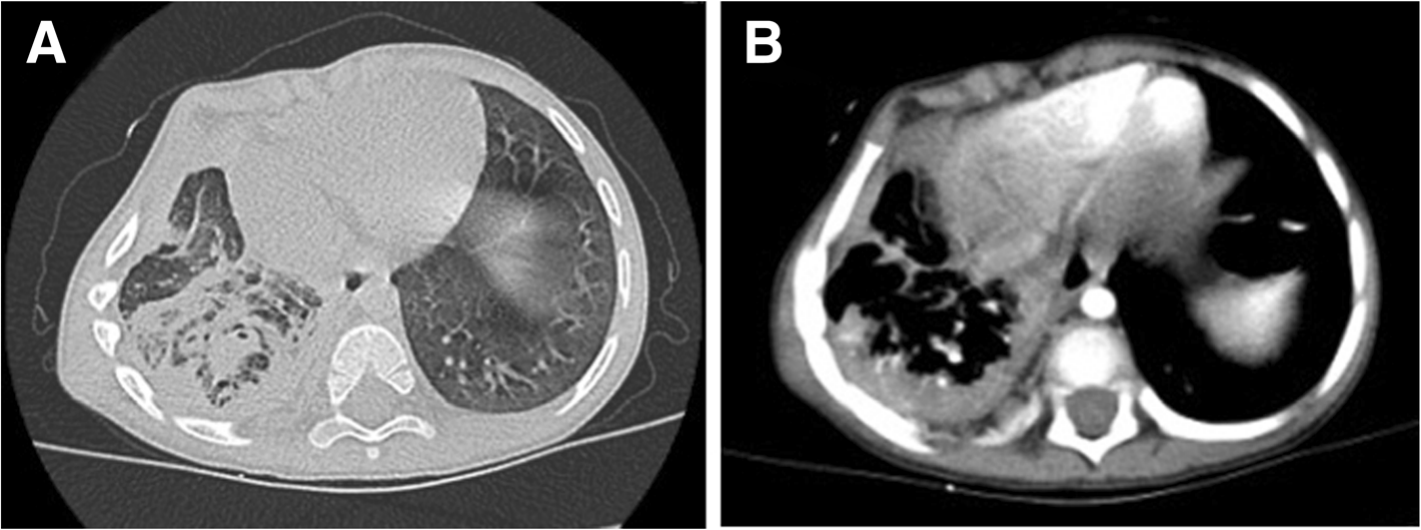
Lung CT showing the presence of 2**a-b** reduced right lung volume, diffuse high-density opacities with patchy shadowing and stripes, many small areas of calcification and cavitation in the lower lobe of right lung, irregular soft tissue of mixed density in the right inferior mediastinum, and calcification in the thickened pleura

A 3-year-old boy presented with a 11-month history of hemoptysis and mild wet cough, with 7 months of intermittent low fever and right thoracic collapse, and 5 months of right-sided chest pain. Chest X-ray revealed right-sided pulmonary consolidation and pleuritis. He was treated with antibiotics, but nonetheless he continued to expectorate bloody sputum or blood (2–5 ml on each occasion), and chest imaging revealed pleural thickening. Pleural decortication was performed and histopathologic analysis revealed fibrous tissue without granuloma. Consequently, he was treated for tuberculosis for 5 months; however, during this time he began to complain of right-sided chest pain. On admission to our hospital, contrast-enhanced CT revealed diffuse high-density opacities with patchy shadowing and stripes, many small areas of calcification and cavitation in the lower lobe of right lung, irregular soft tissue of mixed density in the right inferior mediastinum, and calcification in the thickened pleura (Fig. 2a-b). We made the differential diagnoses of mediastinal teratoma or multifocal myofibroblastoma. On thoracoscopy, we identified a mass tightly adherent to adjacent tissue in the base of the thoracic cavity adjacent to the costospinal angle, and severely adherent, thickened, fibrotic pleura. Most of the right lower lobe was consolidated, necrotic and eroded, while most of the upper and middle lobes were compressed and atelectatic. The tumor and necrotic tissues were excised completely, and air leaks repaired.

#### Case 3

**Fig. 3:**
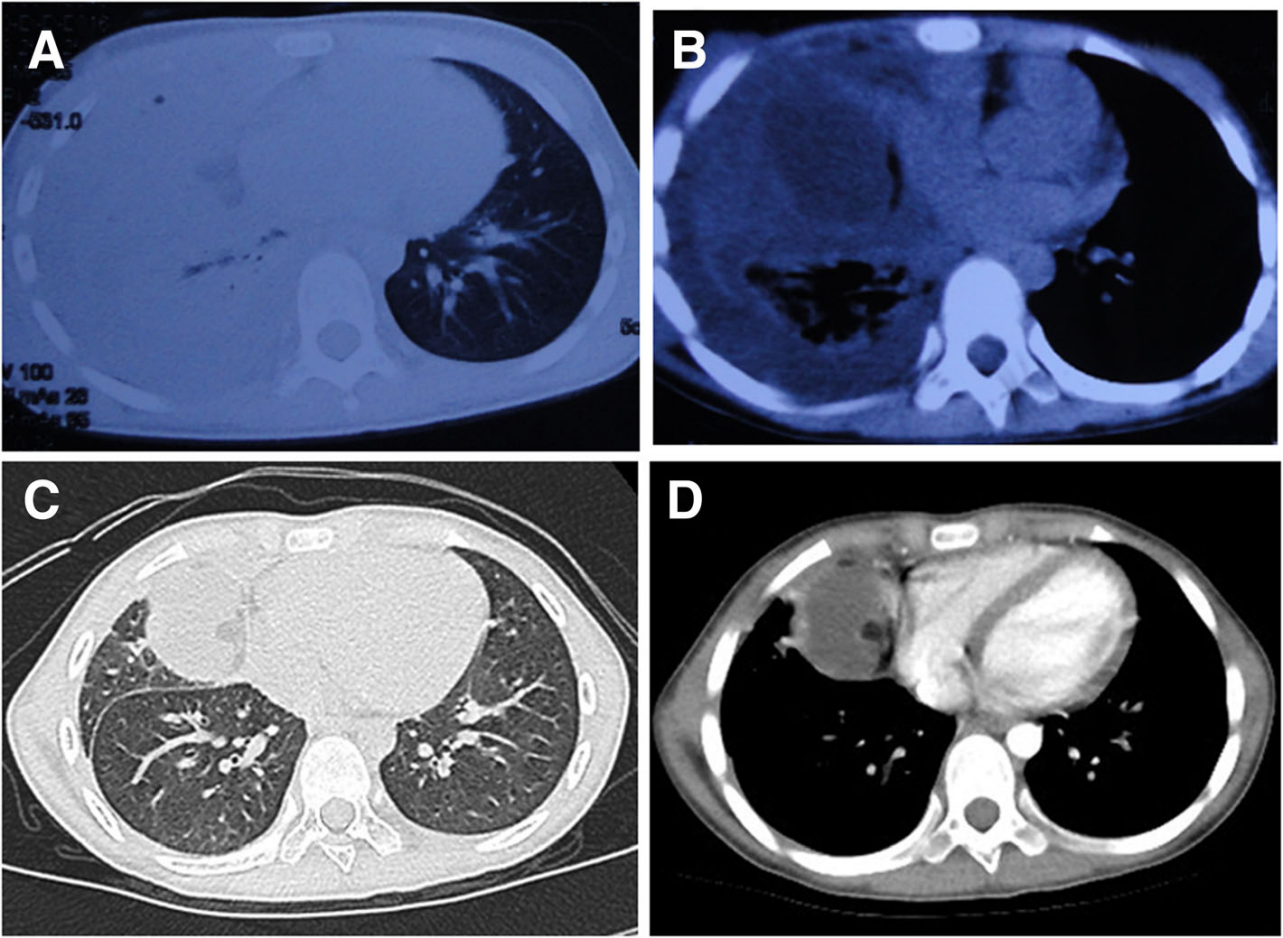
Lung CT showing the presence of 3A right-sided pulmonary consolidation and massive pleural effusion, 3B pachypleuritis and low density mass, and 3C-D a right-sided mass with multiple focal fatty densities adjacent to the heart

A 9-year-old girl presented with a 3-month history of right-sided chest pain and right upper limb pain. In the early stages, she had become suddenly dyspneic after an episode of strenuous exercise. Laboratory investigations showed a WBC count of 22.65 × 10^9^ /l with 77.1% neutrophils, and a serum CRP concentration of 160 mg/l (normal range < 8 mg/l). Thoracic CT revealed right-sided pulmonary consolidation and massive pleural effusion (Fig. 3a). On closed thoracentesis a turbid effusion was drained; it was found to have an elevated leukocyte count of 61,152 × 10^6^/l. An acid-fast stain and bacterial, fungal and mycobacterial cultures of the pleural effusion were negative. Subsequent CT revealed pachypleuritis and a low density mass (Fig. 3b). Pleural decortication was performed and histopathologic analysis revealed fibrous tissue with granuloma. She was successively treated with vancomycin and anti-tuberculosis drugs; however, CT revealed an encapsulated effusion. On admission to our hospital, contrast-enhanced CT revealed a right-sided mass with multiple focal fatty densities adjacent to the heart (Fig. 3c-d). On thoracoscopy, a thymic mass was completely excised. It had been tightly adherent to the right lung, pericardium and diaphragm, all of which were heavily eroded.

All patients were previously healthy and received routine BCG vaccination 3 days after birth. Interferon-gamma release assays in periphery blood, and acid-fast staining in histopathologic analysis were negative in all patients. Histopathologic analysis revealed pancreatic tissue in all patients, and calcification in patients 1 and 2 (Fig. 1e, Table 1). All patients were finally diagnosed with mature cystic-solid mediastinal teratoma (affecting the left upper mediastinum, the right inferior mediastinum and the right anterior mediastinum in patients 1, 2 and 3, respectively). The patients have been followed-up for between 2 years and 6 years. Their recoveries have been uneventful, and there is no evidence of tumor recurrence.

**Table 1:** Demographic and clinical features, histopathologic analysis and prognosis of 3 pediatric patients with mediastinal teratoma

	Patient 1	Patient 2	Patient 3
Gender	Male	Male	Female
Age	5 years	3 years	9 years
Presentation	Hemoptysis, mild wet cough	Hemoptysis, mild wet cough, low fever, chest pain.	Dyspneic,chest pain, upper limb pain
Misdiagnosed diseases	Pneumonia, tuberculosis	Pneumonia, tuberculosis	Pneumonia, empyema, tuberculosis
Tuberculin skin test	An induration of 15 × 15 mm.	An induration of 12 × 12 mm.	An induration of 8 × 9 mm.
Chest imaging in the early stages	Left hilar enlargement	Right-sided pulmonary consolidation and pleuritis	Right-sided pulmonary consolidation and massive pleural effusion
Chest imaging in the middle stages	Cavity within high-density consolidation	Pachypleuritis	Pachypleuritis and a low density mass
Chest imaging at the late stages	High-density opacities occupying the left upper lobe, consolidation with cavitation and calcification adjacent to the mediastinum	High-density opacities with patchy shadowing and stripes, calcification and cavitation in the lower lobe of right lung, irregular soft tissue in the right inferior mediastinum, and calcification in the thickened pleura	Encapsulated effusion
Tumor size(cm)	10 × 9 × 3.5	5 × 3 × 3	5.5 × 5 × 3.5
Histopathologic analysis	Mature pancreatic tissue, gastrointestinal epithelium, cartilage tissue, and sebaceous material within the mass. Chronic cells in some alveolar spaces.	Pancreatic acinar tissue, intestinal epithelium, cartilage tissue, fibrous tissue, sebaceous material, and smooth muscle within the tumor. Proliferative fibrous tissue in the alveolar space and alveolar septa. Necrosis and calcification in pleural specimens.	Pancreatic tissue, digestive tract epithelium, fatty tissue and fibrous tissue within the tumor.
Follow-up	6 years	3 years	2 years
Prognosis	uneventful	uneventful	uneventful

## Discussion

Mediastinal teratoma is mostly seen in young adults, and thymic teratoma is very rare regardless of age; however, patients 1 and 3 had definitive diagnosis of thymic teratoma. A teratoma is not easy to detect when small or not eroding other organs. Mediastinal teratoma with pulmonary and pleural involvement is rare, but the most common symptoms are dyspnea, continuous cough and chest pain [7, 8]. Hemoptysis is a very rare symptom of mediastinal teratoma, and is usually caused by recurrent infection due to lung compression or tumor rupture into the lung [3–5]. In each of these cases, the teratoma had invaded adjacent tissue, especially the lung. In patients 2 and 3, the tumor had also invaded the pleural space, and had caused sudden dyspnea in patient 3. Patients 1 and 2 presented mainly with hemoptysis, but without obvious evidence of infection (especially in patient 1); patients 2 and 3 presented with chest pain.

The classic CT finding of mediastinal teratoma is a round or ovoid, cystic or cystic-solid mass with a clear, smooth outline. The density inside the teratoma is heterogeneous, like water, sebaceous fluid or soft tissue with or without calcified nodules [8]. In patient 1’s early clinical course, the small irregular teratoma lacked clear signs of extension into the lung tissue, and was mistaken for an enlarged hilar lymph node or pulmonary consolidation. However later did lung CT reveal calcification, cavitation and a low-density focus in the irregular mass. In patient 2, chest imaging initially revealed a large lobe consolidation in the right lung and right-sided pleuritis. Only subsequently was calcification identified in the right lower lobe and the thickened pleura, and irregular soft tissue in the right inferior mediastinum. However calcification, cavitation and pachypleuritis were the important features of tuberculosis. Therefore the clinical courses and dynamic imaging findings of patients 1 and 2 were similar to the developmental process of tuberculosis, and to the best of our knowledge there have been no previous reports of mediastinal teratoma presenting in these ways. Patient 3 initially presented with pulmonary consolidation and a massive pleural effusion. In the context of extremely elevated inflammatory markers in serum and pleural effusion, a diagnosis of empyema was reasonable. Even though the mass was only recognized later, it was still easily misdiagnosed as an encapsulated effusion or abscess that had not been completely absorbed. Therefore, mediastinal teratoma can be very difficult to diagnose.

In a few cases, calcification and cavitation are found in teratomas accompanied by lung erosion, resulting in a misdiagnosis of tuberculosis [3, 8], especially in developing countries with a high tuberculosis burden such as China. A case of mediastinal teratoma misdiagnosed as empyema in the early stages has also been reported before, and pleural effusion appeared to be purulent [9]. The tumor shape on chest imaging was extremely atypical in all our patients especially patient 2.

Pancreatic tissue has been identified within mediastinal teratomas before [10, 11]. Sommerlad et al. [6, 12] reported a fistula adjacent to the mediastinum secondary to non-purulent inflammation caused by digestive enzymes, however inflammatory markers were extremely high and the pleural effusion appeared to be turbid in patient 3, which increased the difficulty in diagnosis. It is presumed that pancreatic digestive enzymes are the main cause of tumor rupture and perforation of surrounding structures. In patients 1 and 2, hemoptysis was the chief complaint; the lung tissue adherent to the teratoma was partially eroded in patient 1, and severely eroded, necrotic in patient 2. These findings suggest that the hemoptysis was caused by erosion of small pulmonary vessels by digestive enzymes released by the teratoma. Only a few cases of teratoma rupture into the pleural space have been reported; in addition, these tumors may be associated with atelectasis, and post-obstructive pneumonia [13–17]. Patient 2 presented with atelectasis and secondary collapse of the right lung. Patient 3 had a sudden-onset massive pleural effusion, suggesting that a teratoma rich in sebaceous fluid had ruptured into the pleural space; however, chest imaging at the time also suggested atelectasis or obstructive pneumonia. Pleural biopsies had been performed in patients 2 and 3; the fibrous tissue identified was suggestive of pachypleuritis. Although this was not recognized at the time, this was likely due to pancreatic tissue within the teratoma eroding surrounding structures and causing chronic non-purulent pleural inflammation.

Surgical resection is the only effective way to treat teratoma, especially for mature tumors. All our patients have remained well during follow-up.

## Conclusions

We describe 3 rare cases of mediastinal teratoma misdiagnosed as tuberculosis or empyema. Mediastinal teratoma should be considered as a differential diagnosis in patients with hemoptysis, lung lesions and pleuritis of unknown origin. Calcification and pachypleuritis on chest imaging especially in patients without fever should be highly suspected of mediastinal teratoma. Pleural biopsy sometimes fails to assist in making a definitive diagnosis. Prompt recognition, diagnosis and treatment may reverse not only lung damage, but may prevent other complications.

## Abbreviations

CRP: C-reactive protein
CT: Computed tomography
WBC: White blood cell
